# Utilization of second medical opinions as a function of the payment method

**DOI:** 10.1186/s12913-025-12300-z

**Published:** 2025-02-26

**Authors:** Liora Shmueli, Tuvia Horev

**Affiliations:** 1 Department of Management, Bar-Ilan University, Ramat-Gan, 52900 Israel; 2 Department of Health Policy and Management, Guilford Glazer Faculty of Business and Management, Ben-Gurion University of the Negev, Beer-Sheva, Israel

**Keywords:** Second opinion, Payment track, Co-payment, Reimbursement, Voluntary health insurance

## Abstract

**Background:**

The cost of a medical service and its mode of payment are known to play a role in patient decision-making, as demonstrated by the pioneering RAND Corporation Health Insurance Experiment (HIE). This study explores second medical opinion (SO) utilization by payment tracks- “financial reimbursement track” and “network track” by holders of an optional supplementary health insurance. It estimates SO utilization patterns before and after a “Reimbursement-to-Networks Arrangement” regulatory reform initiated on 2015 that eliminated the reimbursement option for SO consultations specifically involving surgeons, and identifies demographic and clinical predictors associated with each payment track before the reform.

**Methods:**

Retrospective analysis of medical records data, including secondary care visits data from Clalit Health Services, Israel’s largest healthcare provider, and claims data from Clalit’s supplementary insurance. An algorithm based on data from Clalit’s electronic medical records was used to identify SO instances by payment method. Multivariate logistic regression was used to identify characteristics of SO seekers by their payment method.

**Results:**

There was an increase of 28% in patient demand for SO consultations via the supplementary insurance's co-payment track from 2015 to 2017 due to the regulatory reform initiated in 2015. Before this reform, patients from the Arab sector, low socio-economic groups, immigrants, and residents of central geographical areas tended to seek SO via the “network track”. Whereas patients from peripheral areas and Jewish Orthodox tended to seek SOs via the financial reimbursement track.

**Conclusions:**

In line with the RAND Corporation HIE, we show that incentive structures, such as provider payment methods, can explain some of the variability seen in seeking specialists across health plans or payment tracks. Considerations other than cost, such as geographic distance from the service provider, play a role in deciding on the mode-of-payment for a SO. Analyzing utilization patterns can improve the tracking of regulations’ consequences on expenditure, policy, clinical outcomes, and patient satisfaction.

## Callout Box: What is known on this topic/What this study adds.

**Table:** 

Cost and payment methods can influence healthcare decisions (e.g., RAND Health Insurance Experiment)
Limited understanding of how payment tracks within optional health insurance affect seeking second opinions (SO)
Increased demand for SOs via co-payment track after eliminating financial reimbursement option
Socioeconomic factors and location influence preferred payment method for SOs (e.g., Arab sector & low-income preferred network track)
Payment methods are not the only factor—distance to specialists also matters

### Introduction

Patients often opt for a second opinion (SO) from another physician, especially before surgery. The reasons for doing so are varied. Some seek to obtain the best diagnosis, the most appropriate course of treatment, or a more accurate prognosis. Other reasons relate to poor patient-physician communication or lack of trust [1–13]. An interesting aspect that is barely described in the literature is the ability of a patient to pay for a SO or, alternatively, the service being covered or fully/partially reimbursed by the medical insurance company, be it private or public. 

SO payments mechanisms vary across countries and are affected by the local health insurance design, among other things. In most countries with a National Health Insurance (NHI), obtaining a SO prior to surgery is not included in the benefits package. This is the case, for example, in Canada, France and Israel, countries with an NHI, where patients opting for a SO before surgery have to pay a co-payment fee. In contrast, in the U.S., health insurance providers typically require a referral from a primary care physician, will approve an SO only if the doctor is an in-network physician, and the SO's costs will only be partially covered by most health insurance plans [14–16].

Health insurance plans may involve different payment methods for receiving a medical service, all of them necessitating out-of-pocket costs: deductibles, co-insurance and co-pays. A deductible is the amount the insured pays each year for health care services before his/her health insurance begins to share in the cost of covered services. A co-payment is a fixed amount the insured pays for a healthcare service, usually upon receipt of a service. A co-insurance is a portion of the medical cost the insured pays after his/her deductible has been met [17].

In this study, we focus specifically on co-payments, one of the most common cost-sharing methods in both public and private health insurance systems. Co-payments affect the demand for healthcare services, as patients consume less healthcare services when they are required to pay increasing out-of-pocket costs [18, 19]. A majority of studies addressing such considerations found that the requirement for a co-payment reduced patient consultations with specialists [20], while reducing the deductible increases the likelihood of a moral hazard. Moral hazard, as applied in health insurance, refers to the phenomenon where individuals tend to increase healthcare utilization when the out-of-pocket costs are reduced or eliminated by insurance coverage [18]. An interesting question is whether the demand for a service with a co-payment is higher than one with a full-price payment that is subsequently reimbursed. 

In this context, one of the famous studies, “The RAND Corporation Health Insurance Experiment (HIE)”, is still considered a “gold standard” for predicting the likely impact of health insurance reforms on medical spending [21]. This large-scale, randomized experiment was conducted between 1971 and 1982 to study the impact of varying cost-sharing arrangements, including deductibles, co-insurance, and maximum out-of-pocket limits, on healthcare utilization, costs, and health outcomes. The main results of the RAND HIE showed that cost-sharing reduced the use of both highly effective and less effective health services. Specifically, participants with cost-sharing had fewer physician visits annually than those with free care. The study's conclusions encouraged the restructuring of private insurance and helped increase the stature of managed care [21, 22].

The insights from RAND HIE are still relevant today. Incentive structures, such as provider payment methods, can explain some of the variability seen in seeking specialists across health plans and countries. Private health insurance policies whose services involve out-of-pocket payment mechanisms tend to raise co-payment fees and, therefore, increase private health spending by patients. Among Countries in the Organization for Economic Co-operation and Development (OECD), out-of-pocket payments accounted for more than 20% of all national spending on health care in 2017 [23].

In the case of mixed public–private arrangements, under conditions of governmental budgetary distress that affect access to essential public health services, paying co-payment fees within voluntary health insurance (VHI) programs is considered an unfair source of financing due to the widening of gaps for those who cannot afford such co-payments. An ecological study of the U.K. National Health Service’s (NHS) showed that funding of private sector providers of selected elective procedures was associated with a decrease in public provisions and may have contributed to increased inequalities in age and socio-economic in healthcare [24].

Various forms of public/private healthcare mixes are implemented around the globe. In Israel, the main ones blur the boundaries between the public and private healthcare systems [25]. The Israeli healthcare system consists of four non-profit health funds (HFs) that provide a Basic Healthcare Benefits Package to all citizens and permanent residents of Israel, regardless of their ability to pay or demographic differences, each resident must register with one of these non-profit HFs, which operate under the National Health Insurance Law enacted in 1995. These HFs also offer supplemental VHI schemes that demand extra payment, based mainly on age. In 2015, approximately 80% of Israeli households held supplementary health insurance [26, 27]. The supplemental VHI provides complementary services (e.g., alternative medicine, cosmetics), and supplementary services that are not included in the NHI Basic Healthcare Benefits Package, such as a choice of the provider of a SO and selected elective surgeries at private hospitals with co-payment [28–31].

With respect to SO, patients in Israel are legally entitled to obtain SOs according to the Patient Rights Law (1996), but there is no government coverage for this entitlement in the National Health Insurance Law (1995) or any other law. In practice, people in Israel can obtain SOs in several ways. First, through the private sector, by paying completely out-of-pocket directly to physicians or through commercial insurance companies (C-VHI). Second, through a HF supplemental VHI, patients had the option to choose SO between two main payment tracks. The selection of a payment track depended on the specific coverage terms and conditions outlined in the supplementary insurance plans provided by each health fund, which operated in a broadly similar manner.

In the “network track”, patients pay a fixed co-payment fee of approximately US$40 (updated to 2015) directly to a consultant physician affiliated with the supplemental VHI upon receipt of service; up to three SO visits are permitted during a calendar year. In the “financial reimbursement track”, which is typically more expensive than the “network track”, patients can receive partial reimbursement for out-of-pocket SO consultations with a private specialists with a provider not part of the HF’s network (“out-of-network”); the patient pays the full amount to the private consultant and the HFs’ supplementary insurance reimburses the patient for up to 80% of the out-of-pocket cost, limited by an upper bound approximately US$130 per consultation. The cost of a private consultation typically ranges between US$200 and US$500). This reimbursement is quite similar across the four health funds. In 2015, this track was prohibited for SO consultations specifically involving surgeons, requiring patients to utilize the network track instead. Third, Through the secondary care provided by the health funds, by paying a quarterly co-payment for visiting a specialist. Although SO is not included in the NHI Basic Healthcare Benefits Package, patients can consult community-based specialists under the NHI by paying an approximately US$6 copayment per calendar quarter. This coverage applies to consultations with the same specialist during the three-month period.

SOs are costly both to the patient and to the system, and over time, the private–public mix in Israel has widened the gaps in terms of access and freedom of choice. The need to better understand these aspects, along with the need for regulatory intervention, has intensified due to the increase in demand for SOs in Israel, via the supplementary health insurance programs, and the rise in their total net expenditure by more than 50% from 2006 to 2010 [32]. 

Israel’s Ministry of Health and Ministry of Finance have adopted a series of policy reforms concerning the health insurance market designed to strengthen the public health system in Israel and to reduce private cost-sharing for healthcare services [33]. These reforms have had a major impact on the public–private mix in Israel. On November 30, 2015, a regulatory reform initiated by the Israel Ministry of Health prohibited HF supplemental VHI plans from offering reimbursements under the "financial reimbursement track" for SO consultations specifically provided by surgeons. The reform went into effect on July 1, 2016 [34, 35]. This reform was driven by the desire to reduce the national expenditure on health, including the rate of private funding from national expenditure. The Israeli setting can provide as a case study to examine the intensity of regulation on the VHI market since its similarly to European countries, as the VHI is not a duplication of the statutory coverage or substitutive to it [30]. 

The network track offers several advantages for patients, including a fixed co-payment to the provider upon receiving services, reduced out-of-pocket payments, minimized payments at the point of service, and less preoccupation with bureaucratic processes. However, it limits patient choice to in-network providers. For the health insurance, there are also some advantages, such as enabling cost control through a predefined network of providers [36]. In contrast, the reimbursement track provides patients with freedom to choose any physician, including senior specialists. However, the clear advantage of freedom of choice was offset by the relatively high costs [37].

In this study, we aimed to assess how payment method impacts SO utilization. To do so, we compared two payment tracks that involve user charges: the “financial reimbursement track” (reimbursement after payment to the provider) and “network track” (co-payment to the provider upon service receipt). We analyzed and documented the situation before and during the policy change by examining the number of patients seeking SOs via two payment tracks: the "network track" and the "financial reimbursement track”. Additionally, we assessed socio-demographic and clinical predictors associated with each payment track before the regulatory changes, including age, gender, immigration status, socio-economic level, Charlson comorbidity score, peripheral level, and ethnicity. This analysis aimed to better understand the outcomes and the impact of payment methods on SO utilization following the “Reimbursement-to-Networks Arrangement” regulatory reform initiated on 2015 that eliminated the reimbursement option for SO consultations specifically involving surgeons. We believe these findings will enhance understanding of how policy changes influence patients’ reliance on private financing. 

We are unaware of any study that analyzed the utilization of SOs by different payment tracks or that examined the effect of co-payments on the demand for SOs. Only a few studies indirectly addressed patients' characteristics by their insurance type and coverage policy [13, 16] or type of health insurance influence on health care use [38].

### Study objectives

To estimate how many people sought SOs through two payment tracks—"financial reimbursement track" and "network track"—both prior to and during the regulatory changes. Additionally, to identify patients' demographic and clinic predictors associated with each payment track before the regulatory reform initiated on 2015. Such an assessment may serve later as a baseline for future monitoring and analysis of the implications of these policy changes.

## Methods

### Setting

Clalit Health Services, Israel’s largest not-for-profit insurer and healthcare provider, with over 4.5 million members (53 percent market share) and claims data from the supplementary insurance of Clalit Health Services.

### Study design and data collection

We used two datasets linked by a unique patient identifier: (1) Secondary care visits of patients at health fund clinics, including their demographics and clinical characteristics. (2) Compensations for private specialist consultations from the health-fund’s supplementary insurances.

All data were extracted by authorized personnel at Clalit health fund. The extracted data were anonymized by the removal of patient and physician identifying data. The study was approved by the Ethics and Research Committees of Clalit health fund. The data files were transferred to the researchers, who conducted rigorous data quality checks, removing duplicates and outliers, following the ethics rules of the research institute.

### Study population

The target population included Clalit Health Services members aged 21 years and above who sought a SO consultation with consultants in the specialties listed below, through Clalit’s supplementary insurance scheme, between the years 2011–2017. The study period was divided into two phases: prior to the regulatory reform in 2015 and during the reform's implementation (post-2015) to estimate how many people sought SOs through two payment tracks—"financial reimbursement track" and "network track"—both prior to and during the regulatory changes. 

The inclusion criteria for each retrieval year (2011–2017) were: (1) active member of Clalit Health Services (e.g. for 2015, active from 1.10.2014 until 30.3.2016); (2) age 21 years and above; (3) visited at least one specialist during the retrieval year, including 3 months before and after that year, either a secondary care provider in the community or a private consultant claimed from the supplemental insurance, in one of the following specialties: orthopedics; ophthalmology; dermatology; ear, nose and throat (ENT); cardiology; general surgery; urology; gastroenterology; neurology and gynecology.

The exclusion criteria for each retrieval year (2011–2017): Members who only visited a family physician and did not consult a specialist in the above fields; had an indication of treatment or medical procedure visits (i.e., non-clinical consultations); died during the study period; and did not provide a valid patient id number.

### Variables and measurements

The dependent binary variable was the “payment track” used by the patients who sought a SO via the supplementary insurance, based on the contract between the provider and the HF: (1) “Network track,” where the patient pays a fixed co-payment of approximately US$40 (updated to 2015, as of the study reform period) directly to the consultant physician; (2) “Financial reimbursement track,” where the patient consults with a physician authorized by the HF, pays the full cost upfront, and is entitled to reimbursement of up to 80% of the expense, capped at US$130 per consultation. Consultation costs typically range from US$200 to US$500. 

In order to identify patients' demographic and clinic predictors associated with each payment track before the regulatory reform initiated on 2015, the covariates included patient socio-demographics characteristics: (1) age; (2) gender; (3) being an immigrant (i.e., immigrated to Israel after 1989, the years of mass immigration mainly from the former USSR); (4) socio-economic level, defined by the Israel Central Bureau of Statistics, and is based on the financial resources, education, and employment of residence in Israel’s various localities, subdivided into units. Units are clustered into 10 groups: cluster 1 represents the lowest socio-economic level, and cluster 10 the highest socio-economic level. The clusters are divided into 3 groups: low, middle, and high socio-economic level; (5) Charlson comorbidity score (without age), the most widely used clinical index for the evaluation of comorbidities, is based on a weighted index that considers the seriousness of comorbid disease, we grouped the scores into 4 levels: 0, 1–2, 3–4 & > 5 [39]; (6) ‘Periphery’ level, based on the Israel Central Bureau of Statistics, peripheral index, which classifies potential accessibility and proximity of the local authority to the boundary of the Tel Aviv district (cbs.gov.il) The peripheral index includes local authorities ranked by increasing order of centrality, classified into 10 clusters. We grouped these clusters based on their distribution, into 3 groups: peripheral, intermediate, and central; (7) and ethnicity (non-Orthodox Jewish, Orthodox Jewish and Arab sector).

“Second medical opinion” was measured and defined in our previous study using an operative algorithm for identifying SO visits [40]. We defined a ‘second medical opinion’ instance as “consulting with another specialist, in the same specialty, within 3 months of the first consultation in order to get a SO for the same medical problem.” The algorithm identified potential SOs by matching patient consultations with specialists (the “first opinion”) with consultations with another physician (the “second opinion”). 

Each “pair” of a first opinion and a SO could take place in four possible combinations, two of them are relevant to the current study: (1) Both first opinion and SO provided via a supplementary insurance; (2) First opinion provided via the HF in the public system, and the second via a supplementary insurance.

### Statistical analyses

After combining the two datasets into a unified table of SO visits by payment track, we calculated and examined the distribution of patients who sought an SO via the payment track during the years 2011–2017. We decided to examine the characteristics of patients who had consultations during 2015 before the regulatory change.

We produced descriptive statistics of the independent variables for the overall population of members who had a supplementary insurance and sought one SO during 2015 to describe SO utilization by payment method. We checked correlations between the covariates. We compared the socio-demographic characteristics of patients who opted for the “financial reimbursement track” vs. “network track,” using univariate χ2 tests.

A multivariate logistic regression model was examined as an adjusted full model to assess the association between SO utilization by payment method and the covariates. We entered into the multivariate logistic regression only variables that were significant (p < 0.05) in the univariate tests.

Since our analysis included multiple explanatory variables, we applied the backward stepwise elimination method using the Wald test, with a significance level (p-value) threshold of 0.05 for variable elimination at each step. We used SPSS version 23 for the statistical analyses.

### Findings

**Fig. 1:**
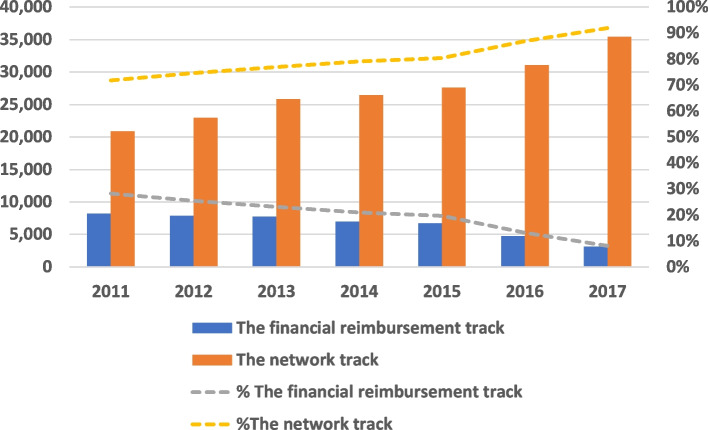
Distribution of patients with supplementary insurance who sought a single second-opinion by payment track (2011–2017)

We observed a decline in the number of patients who sought a SO via the “financial reimbursement track” compared to the “network track” during the years 2011–2017; the sharpest decline took place from 2015 onwards (Fig. 1).

**Fig. 2:**
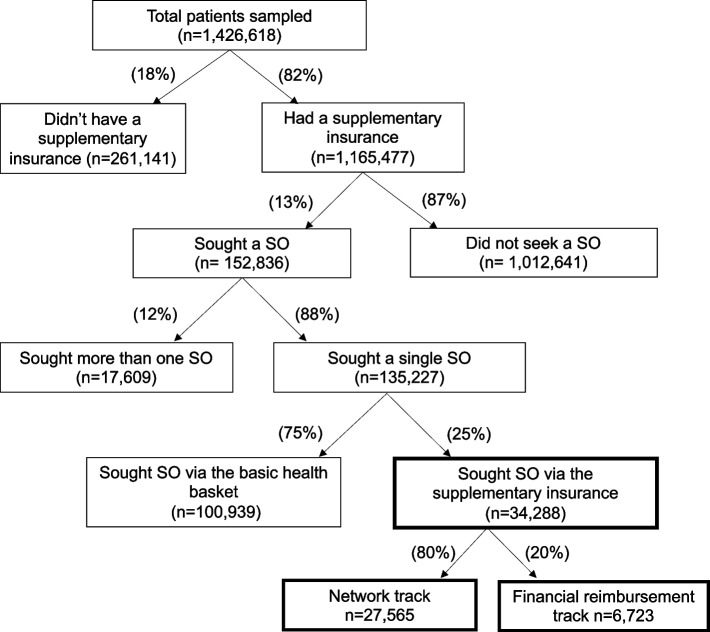
SO by payment track: “financial reimbursement track” vs. “network track” in 2015

The demand for SO consultations via the “network track” increased in parallel to a rise in the number of supplementary insurees during the years 2011–2017. There was a 28% increase in the demand for SOs via the network track of the supplementary insurance from 2015 to 2017 due to the regulatory reform. During this period (2015–2017), the total growth in new supplementary insurance members was only 5% [41] Of the entire sample in 2015, which included 1,426,618 patients, 82% had a supplementary insurance. In this study, we focused on 34,288 patients with supplementary insurance who sought a single SO via the supplementary insurance: either via the “financial reimbursement track” or “network track” (Fig. 2).

### Comparison of seekers of a second opinion by payment track: “financial reimbursement track” vs. “network track

Table 1 presents the characteristics of patients who sought a SO via the “financial reimbursement track” vs. “network track” in 2015. Most of the patients were native-born and senior Israelis, above the age 60 years, of middle socioeconomic status, Jewish, and lived in central residential areas. The univariate analyses showed significant differences in age group, immigration, socioeconomic level, peripheral level, ethnicity, and Charlson Score between those who sought a SO via the “financial reimbursement track” and those who sought one via the “network track.”

**Table 1:** Characteristics of patients who sought a SO via the “financial reimbursement track” vs. “network track” at 2015 (*n* = 34,288)

Characteristic	Financial reimbursement track *n* = 6,723	Financial reimbursement track *n* = 6,723		Network track *n* = 27,564		*p*-value
**Age group (years)**	21–39	1,039	(14.9%)	5,956	(85.1%)	< 0.001
	40–59	1,570	(18.0%)	7,135	(82.0%)	
	60 +	4,114	(22.1%)	14,473	(77.9%)	
**Gender**	Male	3,200	(19.6%)	13,122	(80.4%)	0.993
	Female	3,523	(19.6%)	14,443	(80.4%)	
**Immigration**	Immigrated after 1989	394	(16.2%)	2,045	(83.8%)	< 0.001
	Native-born and seniors	6,329	(19.9%)	25,520	(80.1%)	
**Socioeconomic level**	Low	1,375	(17.5%)	6,492	(82.5%)	< 0.001
	Middle	2,965	(19.9%)	11,941	(80.1%)	
	High	2,374	(20.7%)	9,104	(79.3%)	
**Ethnicity**	Jewish non-Orthodox	6,253	(19.6%)	25,675	(80.4%)	< 0.001
	Jewish Orthodox	254	(28.0%)	652	(72.0%)	
	Arab	216	(14.9%)	1,237	(85.1%)	
**Charlson score**	0	2,358	(17.4%)	11,187	(82.6%)	< 0.001
	1–2	2,770	(20.7%)	10,626	(79.3%)	
	3–4	1,056	(21.9%)	3,756	(78.1%)	
	5–25	539	(21.3%)	1,996	(78.7%)	
**Periphery level**	Periphery of country	1,184	(20.5%)	4,590	(79.5%)	< 0.001
	Intermediate	2,164	(20.8%)	8,264	(79.2%)	
	Centre of country	3,362	(18.7%)	14,636	(81.3%)	
**Total**		6,723		27,564		

**Fig. 3:**
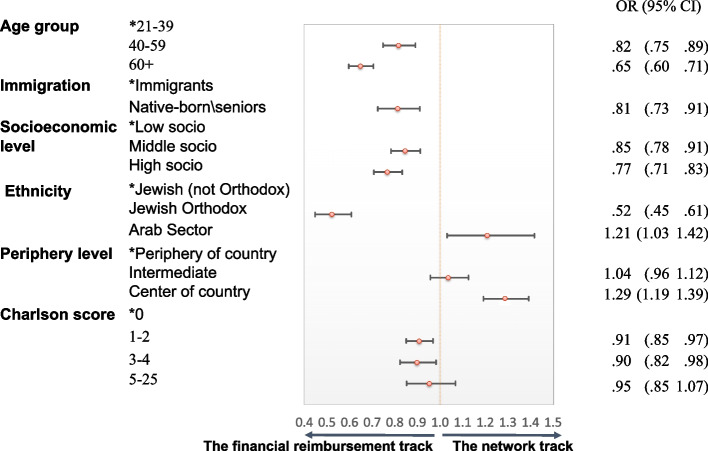
Forest plot of patient characteristics associated with seeking a second opinion by payment method in 2015 (*n* = 34,288). Factors effecting seeking a SO by payment method (“reimbursement” vs. “co-payment”), described by ORs and 95% CIs of the estimated regression coefficients in a multivariate logistic regression model. A predictor that did not enter the final model was “gender.” (*) Reference category

The significant covariates from the univariate analyses that remained significant in the multivariate logistic regression are: age group, immigration, socioeconomic level, ethnicity, periphery level (except Intermediate Level) and Charlson Score (except 5–25 Charlson Score). Patients from the Arab sector (OR = 1.21, 95% CI 1.03–1.42) and from central geographical areas (OR = 1.29, 95% CI 1.19–1.39) tended to seek SOs by paying a co-payment rather than by being reimbursed. HF members who were native-born (OR = 0.81, 95% CI 0.73–0.91), from middle (OR = 0.85, 95% CI 0.78–0.91) and high (OR = 0.77, 95% CI 0.71–0.83) socio-economic levels, Jewish Orthodox (OR = 0.52, 95% CI 0.45–0.61) or had a Charlson score level 3–4 (OR = 0.90, 95% CI 0.82–0.98) tended to seek SOs by the reimbursement method (Fig. 3).

## Discussion

Incentive structures, such as insurance provider payment methods, can explain some of the variability in seeking specialists across health plans or payment tracks. Previous studies explored the role of co-payments in healthcare plans and the tradeoff between the co-payment amount and the utilization of medical care, using the RAND HIE as a basis. The conclusions of the RAND experiment are still relevant in today’s healthcare debate on whether cost-sharing can reduce waste without damaging the quality of healthcare [42–44]. 

Prior research on co-payments in healthcare plans and the RAND HIE produced mixed results. The impact of co-payments on seeking a specialist is complex and varies depending on the specific context and design of the co-payment system. A study conducted in Germany demonstrated that per-period co-payments lead to a decrease in doctor visits, particularly among young adults [45]. Another study, conducted in the U.S., examined the impact of the major Massachusetts health care reform on payments made in the health care sector and provided evidence that it led to increased physician payments in the privately insured market, suggesting a potential link between payment reforms and financial incentives for specialists [46].

The current study examines how an Israeli reform concerning the medical insurance payment structure can impact the selection of specialists by insured individuals. The reform’s elimination of the reimbursement option left individuals only with the option of paying a co-pay in order to visit a service provider from the insurer's network. 

This study demonstrates that before the regulatory change, particular patient profiles, such as those from the Arab sector, low socio-economic, immigrants, and those from central geographical areas, tended to use the "network track" and pay a co-pay when looking for SOs from providers. In contrast, patients who lived in periphery areas and who identified as Jewish Orthodox tended to use the “financial reimbursement” track, even though they had supplemental insurance that allowed them to use the "network track" and pay a lower co-pay. Consistent with previous studies showing that factors related to patients’ choice of out-of-network specialist providers were recommended physician and perceived quality of the provider, in this study, factors related to patient’s characteristics were: patients with high income, higher education, female and poor health status [47, 48].

Several explanations given in the literature refers to the effect of co‐payments on consumers' demand and consuming behavior. One explanation is related to the impact of socioeconomic level indicating that co-payment reduces utilization among patients with a low income [20, 49]. Most of the studies found that co-payment reduces the use of consultations with specialists, among other services [50]. This explains why the demand of some patients’ profiles was lower when it came to paying the full price and receiving partial reimbursement compared to paying a fixed co-payment, especially among low-income groups [20]. In contrast, those with a higher socio-economic status use private health services and consult with more specialists [51, 52]. Another explanation is related to new immigrants, whose socio-economic status and medical costs were lower than native-born residents [53–55].

Another possible explanation may be related to the “supply side” of in-network specialists. Geographical region might also affect the “supply side,” as medical specialists' office location is explained by factors such as financial incentives and proximity to hospitals located in central areas, rather than by the health care needs of the population [56, 57]. This means that patients residing in central areas have greater access to specialists than those who live in peripheral areas. Hence, accessing in-network physicians with only a co-pay for SO might be easier in the geographic center compared to weaker populations in the periphery, creating structural health insurance inequality. Another possible explanation may be related to one of the key characteristics of the "in-network" mechanism is its potential influence on service quality and accessibility. This may inadvertently deter certain population groups, particularly those who prioritize access to highly experienced specialists, from utilizing these services.

### Policy implications

It is important to identify specific patient profile groups and their demand for SO by their payment method and devise appropriate mechanisms for access to in-network physicians providing SOs. Providing data on SO utilization by payment method is important for health policymakers and healthcare providers in order to allocate resources more efficiently.

In terms of resource allocation: planning physicians’ supply is known as a complex and challenging task [58]. This complexity increases when it comes to supply in the private market. There is less control over national resource planning of private resources, especially of private physicians in terms of population density in geographical areas, as opposed to planning resources in the public system. This highlights the need to enhance the supply of in-network physicians, especially in peripheral geographical areas. 

In recent years, policymakers in Israel recommended some regulatory moves in order to deal with the increase in private health insurance [30]. Since 2015, several reforms have been initiated to strengthen Israel's public healthcare system, including the “Cooling-off Period” regulation. This policy, effective November 2017, requires that physicians who treat patients in the public health system (in either hospital or community settings) refrain from providing private consultations to the same patient for a six-month period. Future research should examine the long-term effects of the cooling-off regulation on SO utilization patterns and its broader implications for both public and private healthcare systems.

The increase in private expenditures on SOs widens the gaps between those who can afford to pay privately and those who do not have access to SO or the financial ability to pay co-payments [59, 60] The rise in private spending in the form of cost share is an issue in many OECD countries.

Health policy regarding SOs is a matter of balancing benefits and costs. Certain patient profiles could have benefited from SOs, but do not have access due to the unavailability of specialists in networks in their residential area (especially in peripheral areas, where there is less access to specialists), and the need to allocate resources for that purpose. As specialists tend to reside in central urban areas, it is recommended to incentivize specialists to work in the periphery in order to increase access and availability in those areas. Also, increasing the use of consulting with in-network physicians for SOs by using telemedicine should be considered.

### Limitations of the study

Several limitations should be noted. First, definition of an SO: the definition of an SO as ‘consulting with another specialist, in the same specialty’ relies on the assumption that consequent consultations with different physicians in the same specialty may hint that this consultation was a SO. We previously demonstrated the validity of using this novel tool by achieving a similar percentage with a survey [40]. Second, private consultations: the study lacks data on possible consultations with private physicians that were reimbursed by commercial private medical insurances (i.e., not insurances sold by the HFs themselves) or were not claimed back from any insurance entity. Including such data may provide a more comprehensive analysis of the variation between the beahvior of patients from the public or VHI system vs. the private system. Third, comparison across HFs: it is recommended to explore other HFs’ supplementary insurances (before and after the reform) to more fully comprehend the implications of the regulation. Lastly, Economic Factors**:** while the study identified the regulatory reform as a key factor influencing demand for SO consultations, it did not account for broader economic factors, such as inflation, deflation, or changes in disposable income, which may have also influenced patient behavior. Future research should consider these variables to provide a more nuanced understanding of the drivers behind SO utilization trends.

## Conclusions

In line with the RAND HIE, we show that incentive structures, such as provider payment methods, can explain some of the variability seen in seeking specialists across health plans or payment tracks. Considerations other than cost, such as geographic distance from the service provider, play a role in deciding on the mode-of-payment for a SO. Analyzing utilization patterns can improve the tracking of regulations’ consequences on expenditure, policy, clinical outcomes, and patient satisfaction.

## Data Availability

No datasets were generated or analysed during the current study.
